# Catalytic properties and biological function of a PIWI-RE nuclease from *Pseudomonas stutzeri*

**DOI:** 10.1186/s40643-022-00539-x

**Published:** 2022-05-24

**Authors:** Fei Huang, Xiaoyi Xu, Huarong Dong, Nuolan Li, Bozitao Zhong, Hui Lu, Qian Liu, Yan Feng

**Affiliations:** State Key Laboratory of Microbial Metabolism, School of Life Sciences and Biotechnology, Shanghai Jiao Tong University, Shanghai, 200240 China

**Keywords:** PIWI-RE, Endonuclease, *Pseudomonas stutzeri*, Catalysis, DNA replication

## Abstract

**Background:**

Prokaryotic Argonaute (pAgo) proteins are well-known oligonucleotide-directed endonucleases, which contain a conserved PIWI domain required for endonuclease activity. Distantly related to pAgos, PIWI-RE family, which is defined as PIWI with conserved R and E residues, has been suggested to exhibit divergent activities. The distinctive biochemical properties and physiological functions of PIWI-RE family members need to be elucidated to explore their applications in gene editing.

**Results:**

Here, we describe the catalytic performance and cellular functions of a PIWI-RE family protein from *Pseudomonas stutzeri* (*Ps*PIWI-RE). Structural modelling suggests that the protein possesses a PIWI structure similar to that of pAgo, but with different PAZ-like and N-terminal domains. Unlike previously reported pAgos, recombinant *Ps*PIWI-RE acts as an RNA-guided DNA nuclease, as well as a DNA-guided RNA nuclease. It cleaves single-stranded DNA at temperatures ranging from 20 to 65 °C, with an optimum temperature of 45 °C. Mutation at D525 or D610 significantly reduced its endonuclease activity, confirming that both residues are key for catalysis. Comparing with wild-type, mutant with PIWI-RE knockout is more sensitive to ciprofloxacin as DNA replication inhibitor, suggesting PIWI-RE may potentially be involved in DNA replication.

**Conclusion:**

Our study provides the first insights into the programmable nuclease activity and biological function of the unknown PIWI-RE family of proteins, emphasizing their important role in vivo and potential application in genomic DNA modification.

**Graphical Abstract:**

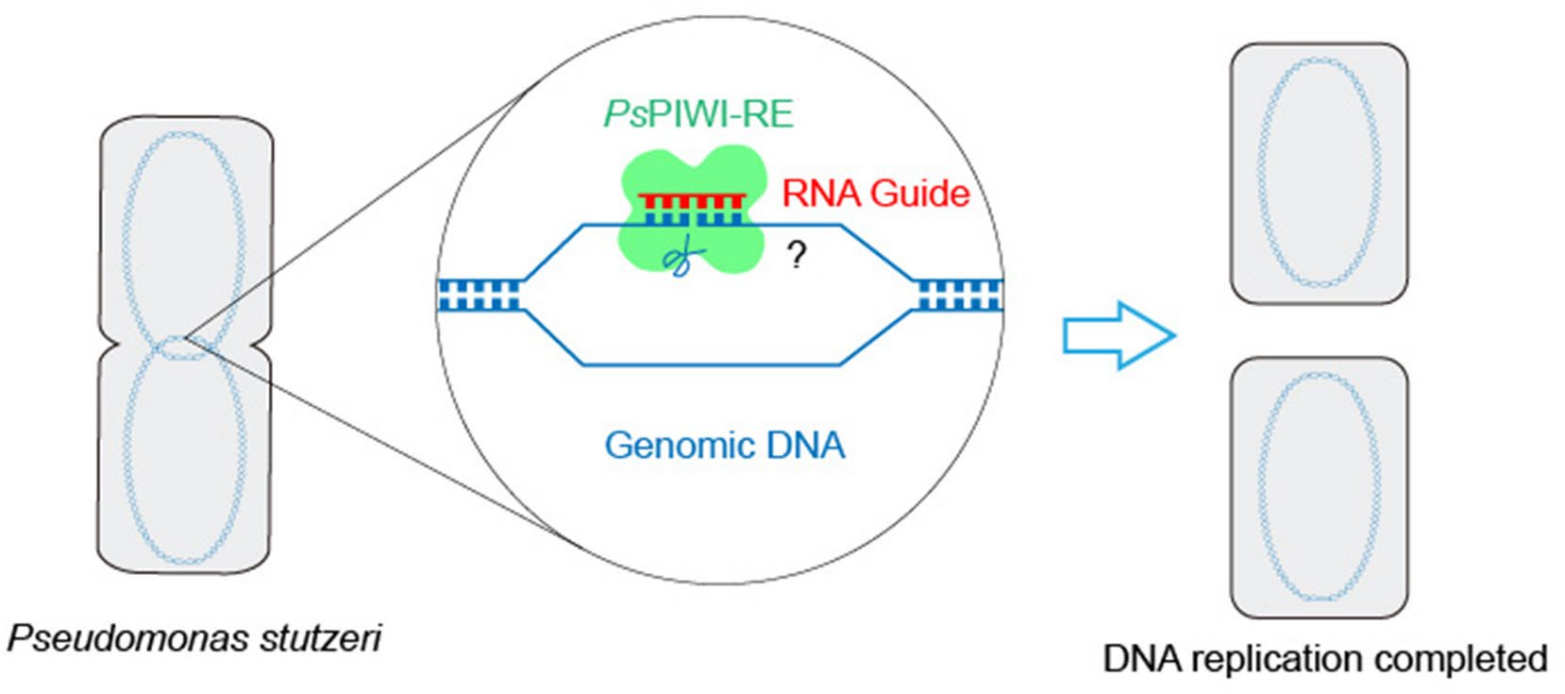

**Supplementary Information:**

The online version contains supplementary material available at 10.1186/s40643-022-00539-x.

## Introduction

Proteins of the Argonaute (Ago) family with a conserved P-element-induced wimpy testis (PIWI) domain play an essential role in several cellular pathways via small RNA and DNA-mediated gene silencing pathways (Swarts et al. 2014b; Xiao and Ke 2016). Extensive studies have indicated that the PIWI domain has a highly similar structure to enzymes in the RNase H family (Song et al. 2004; Song and Joshua-Tor 2006). Ago proteins containing two highly conserved aspartate residues on adjacent β-sheets in the PIWI domain can facilitate targeted cleavage guided by complementary oligonucleotides. Specific cleavage of the target often occurs between nucleotides 10 and 11, complementary to the guide sequence (Wang et al. 2008; Sheng et al. 2014; Willkomm et al. 2017). In contrast to eukaryotic Ago, which performs RNA-guided RNA binding or cleavage in the RNA interference (RNAi) pathway (Hutvagner and Simard 2008), pAgo proteins mostly display DNA-cleavage activity (Swarts et al. 2014a, 2015; Liu et al. 2015; Hegge et al. 2019; Kuzmenko et al. 2019). For instance, the thermophilic Ago from *Thermus thermophilus* (*Tt*Ago) utilises guide DNA (gDNA) with a length ranging from 9 to 36 nt and cleaves ssDNA target strands at temperatures ranging from 20 to 75 °C (Swarts et al. 2014a). Ago from the archaeon *Pyrococcus furiosus* (*Pf*Ago) utilises gDNA with lengths ranging from 15 to 31 nt and is active at temperatures ranging from 60 to 99 °C (Swarts et al. 2015). Owing to their programmable endonuclease activity, thermophilic pAgos have been used in a wide variety of technologies, such as gene mutation enrichment (Song et al. 2019; Liu et al. 2021), nucleic acid detection (Xun et al. 2021), and the creation of artificial restriction enzymes (Enghiad and Zhao 2017). Furthermore, mesophilic Ago proteins, such as Ago from *Clostridium butyricum* (*Cb*Ago), utilise gDNA with a length ranging from 14 to 21 nt and catalyse cleavage of AT-rich double strand DNA (dsDNA) at moderate temperatures (Hegge et al. 2019; Kuzmenko et al. 2019).

The biological function of pAgo is suggested to be related to the host immune response, specifically by interfering with invading DNA in archaea and bacteria (Olovnikov et al. 2013; Swarts et al. 2014a, 2017). Indeed, the presence of *Tt*Ago and *Rhodobacter sphaeroides* (*Rs*Ago) has been reported to decrease plasmid transformation efficiencies in host bacteria (Swarts et al. 2014a; Miyoshi et al. 2016). Recently, it has been reported that *Tt*Ago is involved with DNA replication because nucleic acids associated with *Tt*Ago were derived from the replication terminal regions of chromosomes (Jolly et al. 2020). Similar results were reported for *Cb*Ago (Kuzmenko et al. 2020). Ago from *Natronobacterium gregoryi* (*Ng*Ago) improves the efficiency of homologous recombination and nicks target genomic DNA in *Escherichia coli* (*E. coli*) (Fu et al. 2019; Lee et al. 2021). These results reveal that Ago proteins perform a range of functions in cells, including cell defence and replication.

Bioinformatic analysis has revealed that there are prokaryotic PIWI domain-containing proteins that are distantly related to pAgos; however their functions are unknown (Swarts et al. 2014b; Xiao and Ke 2016). These are known as the PIWI with conserved R and E residues (PIWI-RE) family, and they contain an uncharacterized N-terminal domain instead of the PAZ/N domain found in pAgos (Burroughs et al. 2013). Sequence alignment showed that only a few PIWI-RE proteins contained a complete DEDX catalytic tetrad in their PIWI-like domains, suggesting that endonuclease activity may be retained in these members of the PIWI-RE family. Nevertheless, the function of PIWI-RE family proteins remains unknown. Notably, PIWI-RE-encoding genes often cluster with damage-inducible gene G (DinG) helicases, which belong to the superfamily II (SF-II) DNA helicases with 5′ → 3′ polarity (Burroughs et al. 2013).

In this study, we investigated a PIWI-RE family protein from *Pseudomonas stutzeri* (*Ps*PIWI-RE). First, we systematically characterized the enzymatic properties of this protein, in particular its guide preference and the conditions under which it can catalyse reactions. We then investigated the in vivo function of PIWI-RE by phenotypic analysis and transmission electron microscopy observation of the *piwi-re* knockout mutant. Our work provides the first insights into new Ago family proteins, and shows that *Ps*PIWI-RE could potentially be used as a gene editing tool.

## Materials and methods

### Strains and mutants

For in vivo experiments, the *P. stutzeri* (DSM4166) strain was used, which is hereafter referred to as *P. stutzeri* or wild type. This strain was kindly provided by Prof. Min Lin (Biotechnology Research Institute of Chinese Academy of Agricultural Sciences). Deletion of the entire *piwi-re* locus of *P. stutzeri* was carried out to generate a mutant, which was named *P. stutzeri* △*piwi-re* or △*piwi-re*. All *P. stutzeri* strains were cultivated in Miller’s Luria–Bertani (LB) medium at 30 °C.

To generate *P. stutzeri* △*piwi-re* mutant, the coding region from 5 to 2352 bp of *piwi-re* gene (because the first 4 nucleotides are also consisted of the upstream *dinG* gene) was selected as the deletion region. The upstream and downstream homologous arms of *piwi-re* gene with a length of 1000 bp were amplified using primers of dPSago-F1/dPsAgo-R1 and dPsAgo-F2/dPsAgo-R2, respectively. The two homologous arms were fused by overlap extension PCR, and products were digested with the appropriate restriction enzymes, ligated, and cloned into pK18mobSacB broad-host-range suicide vector to construct pK18mobSacB-△*piwi-re* plasmid. The pK18mobSacB-△*piwi-re* plasmid was transformed into *P. stutzeri* DSM4166 competent cells. Single homologous recombination event was confirmed by selection of kanamycin resistance. Excision of the vector resulting from the second recombination event was achieved on Miller’s LB plates, supplemented with 10% sucrose. Single colonies were screened using colony PCR with primers of det-dPRE-F3 and det-dPRE-R3. The sequences of above primers and homologous arms are shown in Additional file 1: Table S1 and Table S4.

### Protein expression and purification

For *Ps*PIWI-RE protein expression, the gene encoding the full-length sequence for these proteins from *P. stutzeri* was optimised (sequences shown in Additional file 1: Table S4) for *E. coli* codon usage and inserted into the plasmids pET-28a (+). *Ps*PIWI-RE single point mutants (D525A, D610A, R639A, E718A, respectively) were synthesized by GenScript (Nanjing, China). The plasmid was transformed into *E. coli* BL21 (DE3) cells (Novagen). These cells were grown in 1 l Miller LB medium supplemented with kanamycin (50 μg/mL) at 37 °C. To induce expression of *Ps*PIWI-RE, 0.5 mM isopropyl-β-d-thiogalactoside (IPTG) was added. After centrifugation, the collected cells were resuspended in 20 mM Tris–HCl (pH 8.0), 500 mM NaCl, 5 mM MgCl_2_, 2 mM 2-mercaptoethanol, and 10 mM imidazole (lysis buffer) and then lysed by high-pressure homogenisation. Protein purification was carried out using nickel-affinity chromatography, and then desalting was performed using a desalting column (GE Healthcare, USA). The purified protein was diluted to a final concentration of 1.5 mg mL^–1^ in 20 mM Tris–HCl pH 8.0, 500 mM NaCl, 5 mM MgCl_2_, and 2 mM DTT using centrifugal filter devices (Merck KGaA, Germany), and then aliquoted and stored at − 80 °C.

### Fluorescence polarization assays

Fluorescence polarization assays were performed manually in black 96 microplate as previously described (Miyoshi et al. 2016). Each well (100 μL) contained 5 nM 3′-6-FAM-labelled oligonucleotides and varied concentrations (0–1.5 μM) of *Ps*PIWI-RE in reaction buffer (20 mM Tris–HCl (pH 7.5), 250 mM NaCl, and 5 mM MgCl_2_) for 30 min at 37 °C. The polarisation values were measured with excitation at 494 nm, emission at 522 nm in a SpectraMax M5e reader (Molecular Devices, CA, USA). The data were fitted to a nonlinear regression curve using the one-site-specific binding function in GraphPad Prism 8. All experiments were performed in triplicate.

### Electrophoretic mobility shift assay (EMSA)

Varying concentrations of *Ps*PIWI-RE (1–3 μM) were incubated with 1 μM 3′-6-FAM-labelled oligonucleotide chains in 10 μL binding buffer containing 20 mM Tris–HCl (pH 8.0), 150 mM NaCl, 2 mM DTT, and 5 mM MgCl_2_ for 15 min at 25 °C. After incubation, 1 μL 10× dye solution containing 50% glycerol and 0.1% bromophenol blue was added to each reaction mixture. The reaction samples were fractionated by electrophoresis (120 V, 1 h) on 8% native polyacrylamide gels (39:1 acrylamide/bisacrylamide) with 5 mM MgCl_2_ in TBE buffer, and fluorescence in the blue visible light region (wavelength 475 nm) was detected. All experiments were performed in triplicate.

### DNA cleavage assay

In the DNA cleavage assay, 5 μM purified *Ps*PIWI-RE was mixed with guides of either synthetic ssDNA or single-stranded RNA (ssRNA) (Additional file 1: Table S1) in a 5:1 ratio (protein: guide) in 10 μL reaction buffer containing 20 mM Tris–HCl (pH 8.0), 150 mM NaCl, 2 mM DTT, and 10 mM MgCl_2_ and incubated for 15 min at 37 °C. Then, 3′-6-FAM-labelled ssDNA or ssRNA targets (Additional file 1: Table S1) were added to a final 25:5:1 ratio (protein: guide: target) and incubated for 2 h at 95 °C. After adding 10 μL of 2 × loading buffer (95% formamide, 0.5 mM EDTA, 0.025% bromophenol blue, 0.025% xylene cyanol FF, and 0.025% SDS), the samples were resolved on 16% denaturing polyacrylamide gels. Unless otherwise specified, the gels were photographed in the blue visible-light region (wavelength 475 nm).

For measuring temperature effect, 5 μM *Ps*PIWI-RE and 1 μM ssRNA guide were mixed in a 5:1 ratio (protein: guide) and pre-incubated at varied temperatures (20–85 °C) for 15 min. Next, target DNA was added to a final 25:5:1 ratio (protein: guide: target) and the sample was incubated for 2 h at the same temperature. Quantitative analyses of electrophoretic separations of oligonucleotides were performed using the Gel-Pro Analyzer 4 software (Media Cybernetics, USA). Triplicate results were used, and the grayscale of the product band divided by the grayscale of the product and substrate bands was the shearing efficiency. All experiments were performed in triplicate.

### Phenotypic analysis

For the ciprofloxacin resistance assay, once the wild-type and △*piwi-re* strain cultures reached an optical density (OD)_600_ of 0.5, they were divided into two groups. One group was treated with 5 μM ciprofloxacin for 30 min and the other was set as the control. The cultures were diluted and spread onto LB plates. After overnight culturing, the number of plaques was counted. To calculate the survival ratio, the number plaques in the ciprofloxacin-treated groups was divided by the number plaques in the control group. For spot plating assays, after a five-fold serial dilution of cultures, 5 μL of each dilution was transferred to a Miller LB plate and cultured for two days at 30 °C. The plates were photographed to analyse the growth status. For transmission electron microscopy observation, wild-type and Δ*piwi-re* strain cultures were treated with or without 5 μM ciprofloxacin for 6 h, and collected for transmission electron microscopy (Tecnai G2 Spirit BIOTWIN, FEI, USA). For strain length statistics, the lengths of 50 cells in each group were counted using Digital Micrograph software (Gatan, USA), The data were analysed in GraphPad Prism 8. All experiments were performed in triplicate.

For growth curve analysis, *P. stutzeri* wild-type and △*piwi-re* strains were grown overnight at 30 °C in LB medium. 1 mL overnight cultures were transferred to 100 mL fresh LB medium, and cultured at 30 °C, 220 rpm. The OD_600_ was measured every 1 h during 16 h. Experiments were performed in triplicate. Growth curves were plotted from triplicate data, using Graphpad Prism 8.

## Results and discussion

### Structure prediction of *Ps*PIWI-RE

**Fig. 1:**
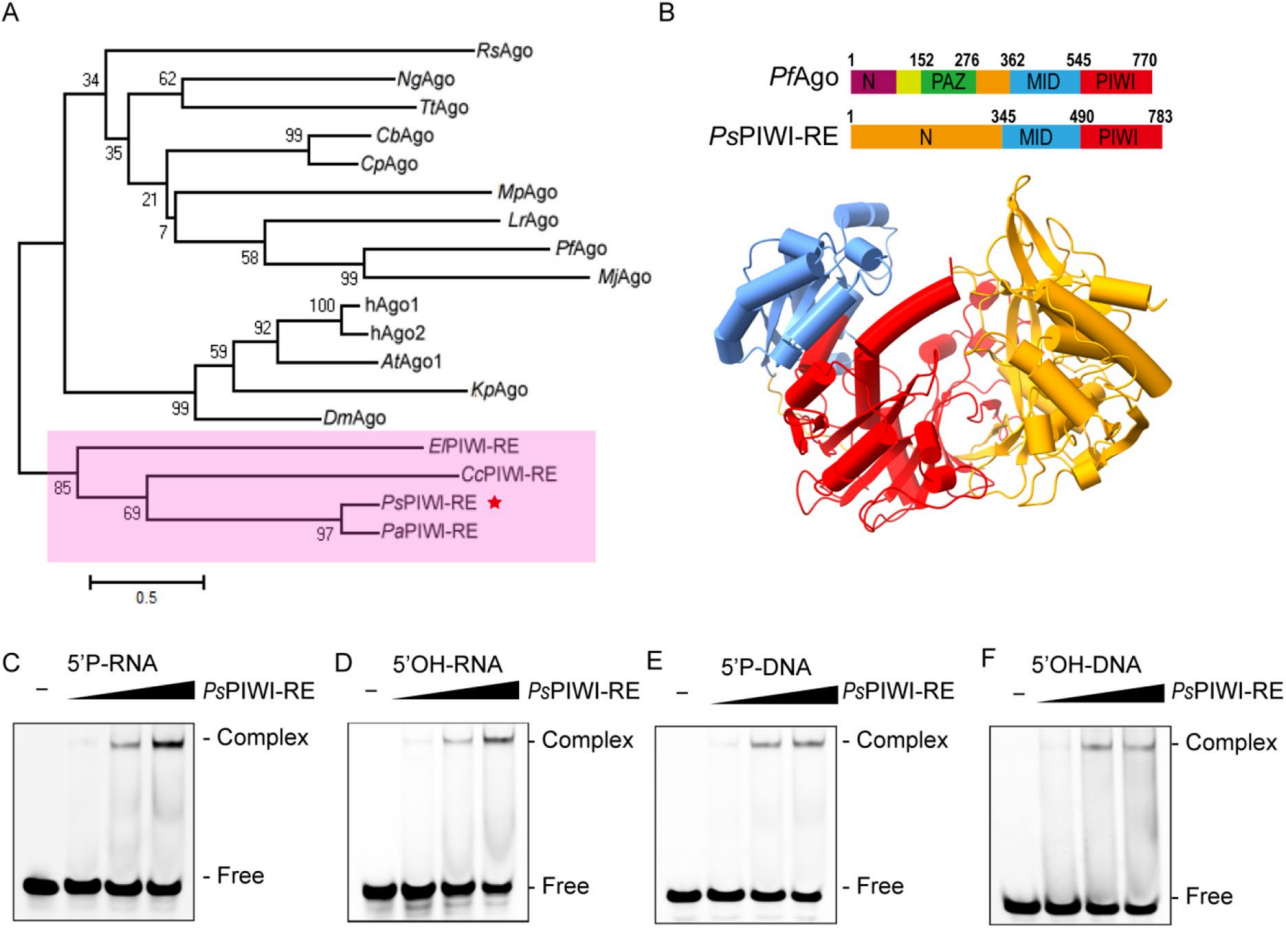
*Ps*PIWI-RE is a distinct group of PIWI-RE with a unique N-domain that binds various guide strands. **A** Phylogenetic analysis of pAgo and PIWI-RE proteins. Phylogenetic tree generated by the Maximum Likelihood method of MEGA4. We computed 1000 replicates for bootstrap support. The pink area represents the PIWI-RE family and the red star indicates *Ps*PIWI-RE. **B** Domain architecture analysis of *Ps*PIWI-RE predicted by AlphaFold 2 reveals that *Ps*PIWI-RE contains a unique N-domain, MID domain, and PIWI domain. **C**–**F** Binding affinities of four types of different guides to *Ps*PIWI-RE measured by EMSA. 1 μM each of 21 nt 5′ phosphorylated (5′P) DNA, 5′hydroxyl (5′OH) DNA, 5′phosphorylated (5′P) RNA, and 5′hydroxyl (5′OH) RNA were incubated with varying concentrations of the *Ps*PIWI-RE protein (1–3 μM) and are shown in figure parts **C**–**F**, respectively. Each guide strand was modified by 3′-6-FAM. “-”: reaction without *Ps*PIWI-RE. The accession number of mentioned proteins and oligonucleotide sequences of the guide strands are shown in Additional file 1: Table S1

PIWI-RE proteins with uncharacterized N-terminal domains were found to be highly divergent from Ago proteins (Fig. 1A). Since enzyme function is highly correlated with structure, we first predicted the structure of *Ps*PIWI-RE using the deep-learning neural network AlphaFold 2 (Jumper et al. 2021). The predicted structure of *Ps*PIWI-RE contained conserved PIWI and middle (MID) domains (Fig. 1B). Unlike *Pf*Ago and *Rs*Ago, the fold associated with nucleic acid binding was positioned away from the nuclease active site (Fig. 1B; Additional file 1: Fig. S1A, B). The predicted N-terminal domain of *Ps*PIWI-RE contains a unique PAZ-like domain, in which β-sheet 6 forms a hydrogen bond with β-sheet 12, which makes this domain more stable. Moreover, the N-terminal domain contained more α-helices than previously reported Agos. This unique structure suggests that *Ps*PIWI-RE performs a unique catalytic function.

### Types of guide oligonucleotides bound by *Ps*PIWI-RE

To predict the types of nucleic acid that act as guide molecules for *Ps*PIWI-RE, an electrophoretic mobility shift assay (EMSA) was performed with four types of 21 nt single-stranded oligonucleotides: 5′-phosphorylated (5′P) DNA, 5′-hydroxyl (5′OH) DNA, 5′-phosphorylated (5′P) RNA, and 5′-hydroxyl (5′OH) RNA (Additional file 1: Table S1). The results showed that *Ps*PIWI-RE bound to all four types of guide strands (Fig. 1C). Furthermore, to quantitatively analyse the binding affinity between *Ps*PIWI-RE and the different guide strands, a fluorescence polarisation assay was set up to measure the equilibrium dissociation constant (*K*_D_) using a 21 nt DNA/RNA guide with 3′ FAM. We found that the 5′P and 5′OH 21 nt DNA bound more tightly to *Ps*PIWI-RE than the 5′P and 5′OH 21 nt RNA (Table 1; Additional file 1: Fig. S2). We then quantified *K*_D_ values for the 21 nt 5′-OH-RNA guide with different 5′ nucleotides. The binding affinity of the RNA guide with all 5′ bases was almost the same as that of *Ps*PIWI-RE, except for 5′C (Table 1; Additional file 1: Fig. S2), suggesting that *Ps*PIWI-RE displays no obvious preference for the 5′-end nucleotide of the guide.

**Table 1:** Equilibrium dissociation constant (*K*_D_) values of the binding affinity of *Ps*PIWI-RE to DNA and RNA oligonucleotides

Oligonucleotides	*K*_D_ (nM)
5′P-DNA(5′T)	24.61 ± 1.57
5′P-RNA(5′U)	64.44 ± 3.53
5′OH-DNA(5′T)	19.71 ± 2.52
5′OH-RNA(5′U)	86.50 ± 16.68
5′OH-RNA(5′A)	88.42 ± 14.83
5′OH-RNA(5′C)	152.30 ± 29.00
5′OH-RNA(5′G)	86.74 ± 11.02

The *K*_D_ values were measured using a fluorescence polarization assay with various nucleic acids

### Programmable endonuclease catalysis of *Ps*PIWI-RE

**Fig. 2:**
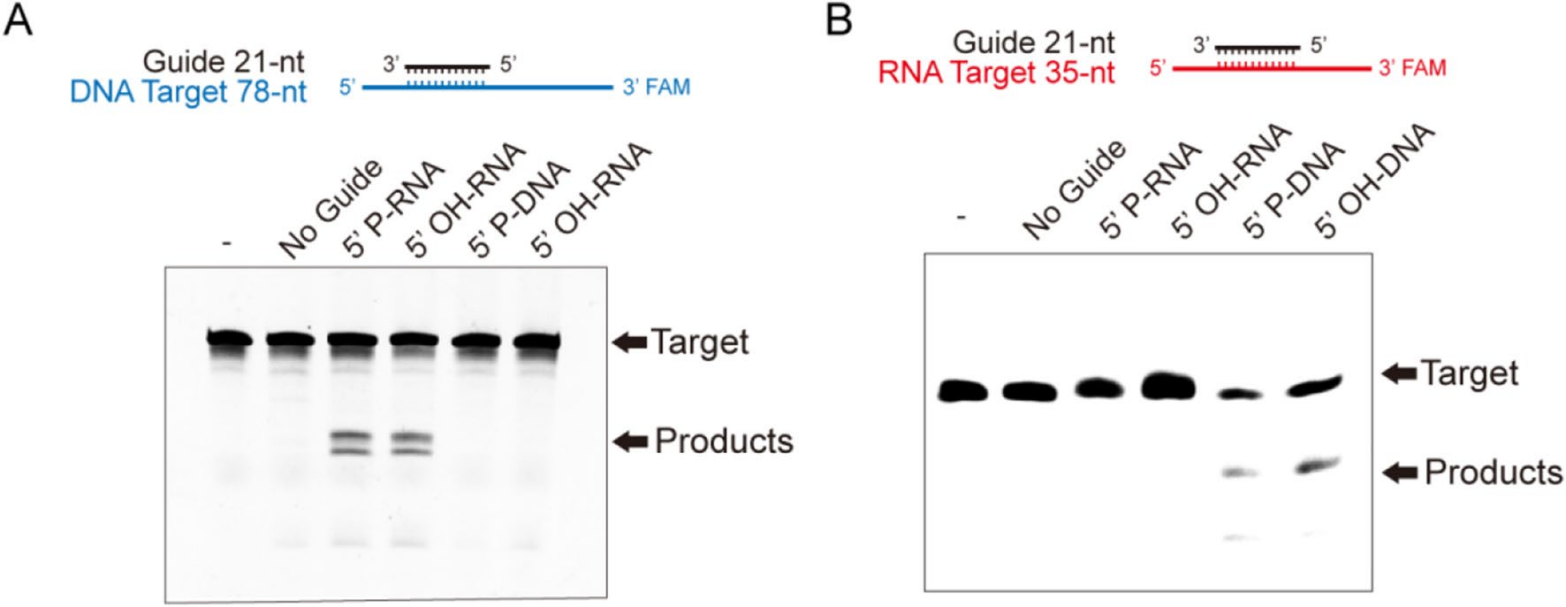
*Ps*PIWI-RE possesses RNA-guided DNA cleavage and DNA-guided RNA cleavage activity. **A** DNA cleavage assays of *Ps*PIWI-RE were examined in the presence of 21 nt guides with different 5′-terminal modifications, which were as follows: 5′ phosphorylated (5′P) DNA, 5′hydroxyl (5’OH) DNA, 5′ phosphorylated (5′P) RNA, and 5′ hydroxyl (5′OH) RNA. A 78 nt single stranded DNA (ssDNA) with a 3′ FAM label was the target sequence. No Guide: reaction without guides. “–”: reaction without guides and *Ps*PIWI-RE. **B** RNA cleavage assays of *Ps*PIWI RE were examined in the presence of 21 nt guides with different 5′-terminal modifications, which were as follows: 5′ phosphorylated (5′P) DNA, 5′ hydroxyl (5′OH) DNA, 5′ phosphorylated (5′P) RNA, and 5′ hydroxyl (5′OH) RNA. A 35 nt single-stranded RNA (ssRNA) with a 3′ FAM label was the target. The oligonucleotide sequences of the guide strands and target strands are shown in Additional file 1: Table S1

Since *Ps*PIWI-RE binds to four types of guide strands, as shown by the above EMSA results, we tested the sequence-specific cleavage of RNA or DNA target strands with 5′P or 5′OH guide oligonucleotides. Unexpectedly, only 21 nt 5′P or 5′OH RNA, but not 5′P or 5′OH DNA, guided *Ps*PIWI-RE to cleave the complementary ssDNA targets (Fig. 2A). We also observed that multiple DNA fragments were produced by *Ps*PIWI-RE cleavage (Fig. 2A). This was also reported to occur with *Methanocaldococcus jannaschii* Ago (*Mj*Ago) and *Marinitoga piezophila* Ago (*Mp*Ago) (Kaya et al. 2016; Willkomm et al. 2017). Additionally, 21-nt 5′P or 5′OH DNA, but not 21-nt 5′P or 5′OH RNA, guided *Ps*PIWI-RE cleavage of complementary RNA targets (Fig. 2B). The RNA-guided DNA cleavage and DNA-guided RNA cleavage activity of *Ps*PIWI-RE suggest that the nucleic acid-binding pocket of *Ps*PIWI-RE may only be suitable for binding DNA/RNA hybrid strands.

Our results showed that the efficiency of *Ps*PIWI-RE-mediated DNA/RNA cleavage is lower than that of DNA-guided DNA cleavage and is mediated by pAgo proteins, such as *Cb*Ago (Hegge et al. 2019; Kuzmenko et al. 2019) (Fig. 2A). The conserved oligonucleotide-guided DNA cleavage activity suggests that the N-terminal domain of *Ps*PIWI-RE may perform a similar function as the PAZ and N-terminal domains of pAgos. However, in the PIWI-RE subfamily, only a few proteins contain key catalytic residues in their endonuclease domains (Burroughs et al. 2013), suggesting that the PIWI-RE subfamily plays a role in DNA binding rather than DNA cleavage, similar to *Rs*Ago, which exhibits RNA-guided DNA binding activity (Miyoshi et al. 2016). As an RNA-guided DNA endonuclease, *Ps*PIWI-RE is an excellent candidate for biotechnological applications, such as genome editing (Hegge et al. 2017). It is a better candidate than DNA-guided pAgos, because RNA guides can be easily obtained by transcription.

### Characterization of DNA cleavage by *Ps*PIWI-RE

**Fig. 3:**
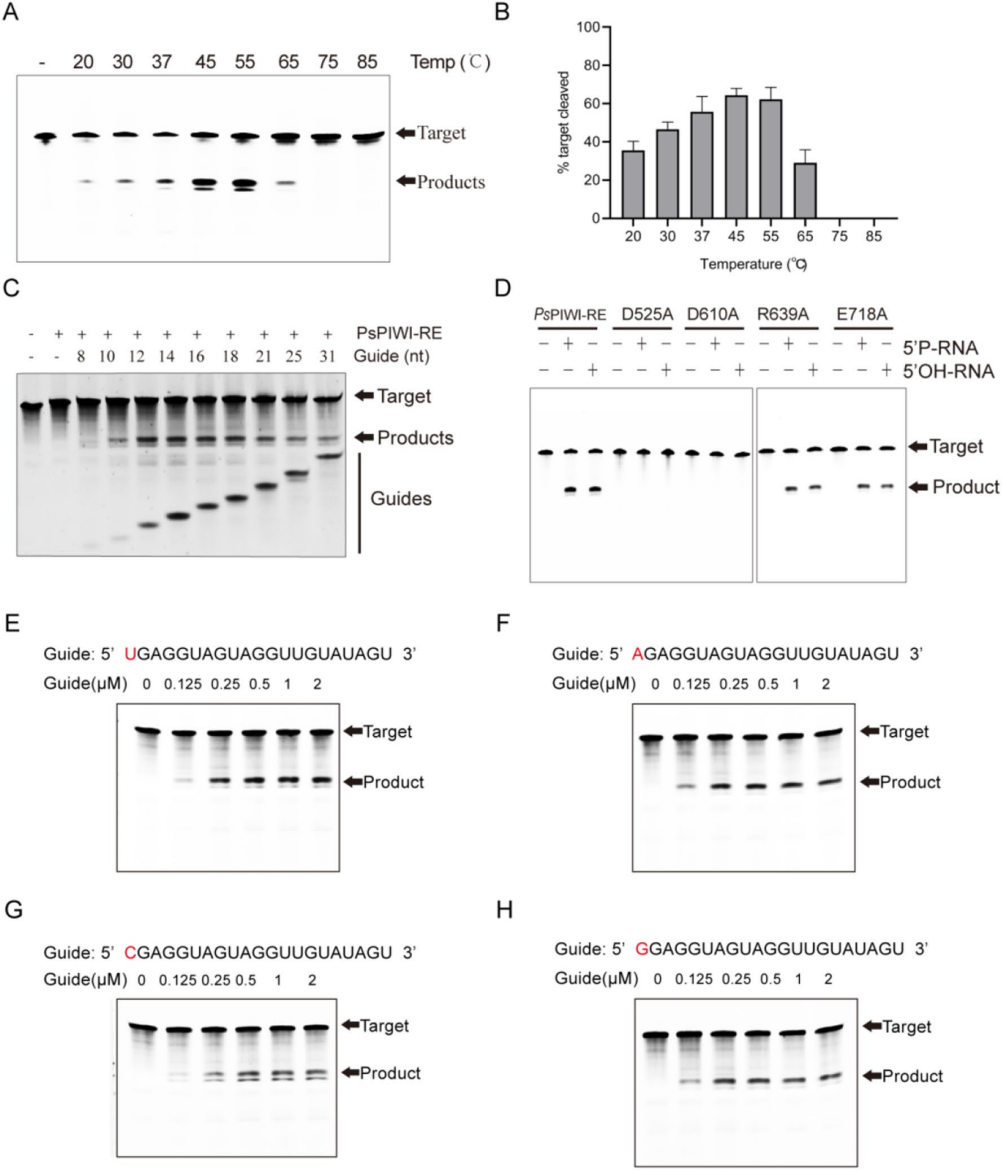
Effect of a range of reaction conditions on RNA-guided DNA cleavage activity of *Ps*PIWI-RE. **A** Temperature dependence of the DNA target cleavage reaction with a 5′ phosphorylated (5′P) RNA guide. “–”: without *Ps*PIWI-RE. **B** Quantitative analysis of the cleavage efficiency of *Ps*PIWI-RE at different temperatures. Histogram were plotted from triplicate data. Mean values ± SD are shown, *n* = 3. **C** Dependence of the efficiency of DNA cleavage on the length of guide RNA. **D** DNA cleavage assays of *Ps*PIWI-RE and mutants. *Ps*PIWI-RE was loaded with a 21 nt guide RNA and was incubated with a 78 nt single stranded DNA (ssDNA) target in a 25:5:1 molar ratio (protein:guide:target). The target cleavage took place at 37 °C for 4 h with 10 mM Mg^2+^. Products were resolved on a 16% denaturing polyacrylamide gel. **E**–**H** Effect of the variation of the 5′ end nucleoside of the guide RNA on *Ps*PIWI-RE cleavage efficiency. 0–2 μM of 21 nt RNA guides containing a different 5′ end nucleoside were incubated with *Ps*PIWI-RE, followed by 78 nt target DNA. The 5′ nucleoside modifications were as follows: 5′ phosphorylated (5′P) DNA, 5′ hydroxyl (5′OH) DNA, 5′ phosphorylated (5′P) RNA, and 5′ hydroxyl (5′OH) RNA. Products of the reactions were resolved on denaturing polyacrylamide gels

To identify the optimum temperature for *Ps*PIWI-RE cleavage, we performed DNA cleavage experiments at temperatures ranging from 20 to 85 °C. The results showed that RNA-guided *Ps*PIWI-RE could cleave ssDNA at temperatures ranging from 20 to 65 °C, with an optimum temperature of 45 °C (Fig. 3A, B). A variety of guide strand lengths was also used, ranging from 8 to 31 nt, and it was found that 10 nt was the minimum length required for *Ps*PIWI-RE activity (Fig. 3C). This distinguishes *Ps*PIWI-RE from DNA-guided pAgos, such as *Cb*Ago, which utilise guide DNAs lengths ranging from 14 to 21 nt (Hegge et al. 2019).

Based on bioinformatic analysis, the active site of *Ps*PIWI-RE includes catalytic residues D525 and D610, and the conserved residues R639 and E718 (Additional file 1: Fig. S2C, D). To confirm the function of these residues, mutants D525A, D610A, R639A, and E718A were constructed and DNA cleavage activity was assessed. D525A and D610A mutants abolished the cleavage activity of *Ps*PIWI-RE, while R639A and E718A had similar activity as wild-type (Fig. 3D). Hence, D525 and D610 were confirmed to be catalytic residues; and it was determined that the conserved residues R639 and E718 were not necessary for the catalytic activity of *Ps*PIWI-RE. Additionally, *Ps*PIWI-RE utilized 5′OH RNA guides with all 5′ nucleotides to cleave the DNA target strand, suggesting that *Ps*PIWI-RE does not exhibit a preference for any particular 5′ terminal nucleotide (Fig. 3E–H).

### Deletion of* piwi-re* affects cell division in *P. stutzeri*

**Fig. 4:**
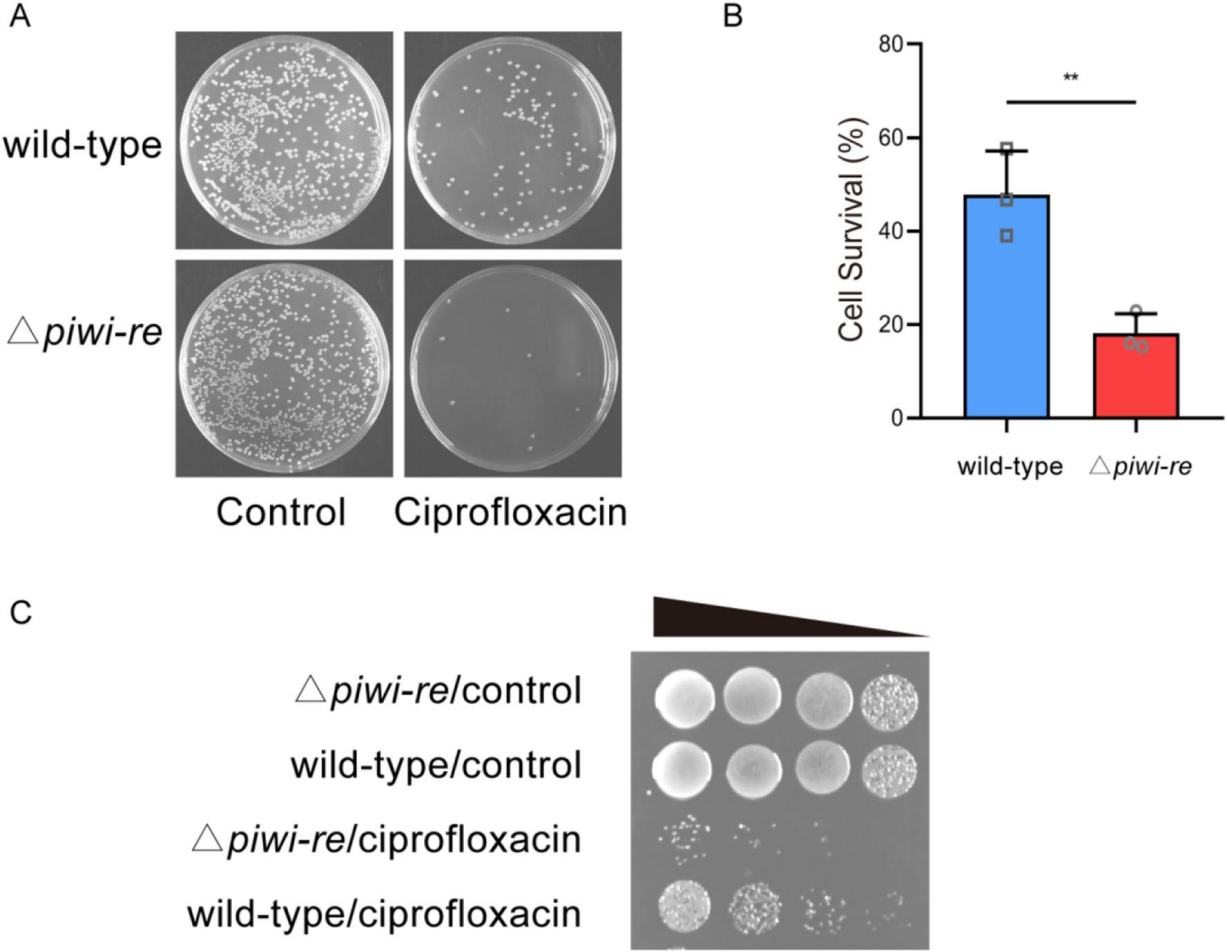
Effects of ciprofloxacin on the survival rates of wild-type and △*piwi-re* strains of *P. stutzeri*. **A** Growth status of wild-type and △*piwi-re* strains after ciprofloxacin treatment. The control group was not treated with ciprofloxacin. **B** Survival rate of wild-type and △*piwi-re* strains after ciprofloxacin treatment. Mean values ± SD are shown (*n* = 3, **p = 0.0016). The *p* value was calculated using an unpaired Student’s *t*-test. **C** Effect on growth state of wild-type and △*piwi-re* strains after treatment with ciprofloxacin and serial dilution

**Fig. 5:**
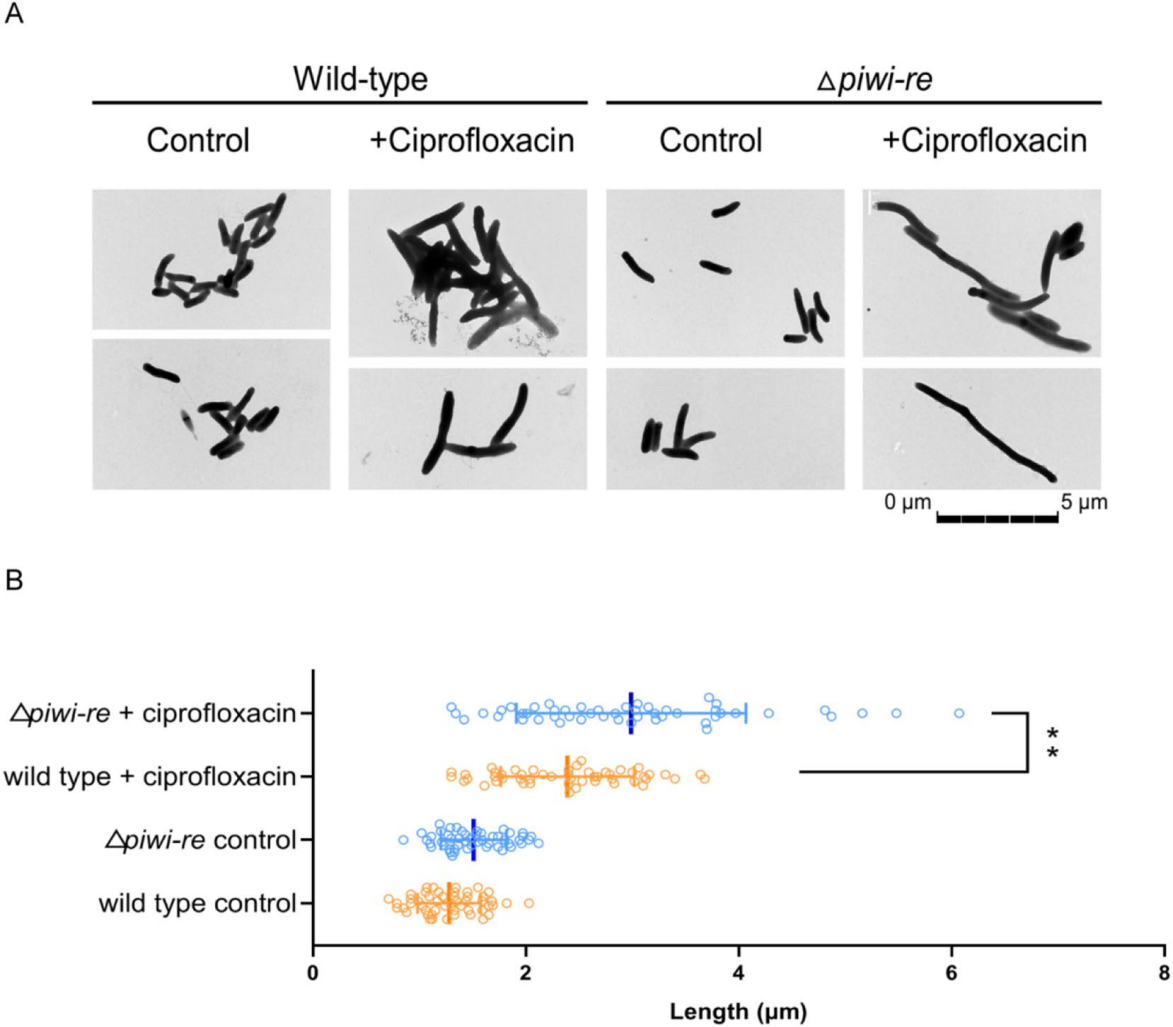
Effects of ciprofloxacin on the morphology of wild-type and △*piwi-re* strains of *P. stutzeri*. **A** The growth of wild-type *P. stutzeri* and △*piwi-re* strains in the presence of ciprofloxacin. The wild-type and △*piwi-re* strain cultures were grown to an optical density (OD_600_) of 0.5 before being divided into two groups. One group was treated with 5 μM ciprofloxacin for 6 h, while the other group was not treated with ciprofloxacin and was the control. Experiments were performed in triplicate and representative transmission electron microscope images are shown. **B** Quantitative analysis of the effect of ciprofloxacin on strain length of wild-type and △*piwi-re* strains of *P. stutzeri*. The lengths of 50 cells in each group were counted using Digital Micrograph software (Gatan, USA), The data were analyzed in GraphPad Prism 8. Mean values ± SD are shown (*n* = 50, **p = 0.0010). The *p* value was calculated using an unpaired Student’s *t* test

To explore the biological function of *Ps*PIWI-RE, a mutant *P. stutzeri* strain (△*piwi-re*) with *Ps*PIWI-RE deletion was constructed using a homologous recombination and then confirmed by PCR and real-time PCR (Additional file 1: Fig. S3A, B). Growth curve analysis of *P. stutzeri* wild-type and △*piwi-re* strains showed that no obvious difference in cell growth between the wild-type and △*piwi-re* strains (Additional file 1: Fig. S4). However, we found that after treatment with ciprofloxacin (a DNA replication inhibitor), colony counts were significantly lower for the Δ*piwi-re* strain than for the wild-type strain (Fig. 4A). The survival rate of the wild-type strain was approximately 15 times greater than that of the Δ*piwi-re* strain after treatment with ciprofloxacin (Fig. 4B). Similar results were observed in the spot-plating assays (Fig. 4C). Ciprofloxacin inhibits DNA replication by inhibiting bacterial DNA topoisomerase and DNA-gyrase. (Lewin et al. 1991; Pan et al. 1996). Inhibition of DNA replication by ciprofloxacin directly results in the arrest of cell division in some bacteria (González et al. 2018). To analyse the effect of deletion of *piwi-re* on the cell division of *P. stutzeri*, we furthermore observed the morphology of wild-type and Δ*piwi-re* strains with or without ciprofloxacin treatment by transmission electron microscopy. The results showed that the Δ*piwi-re* strain formed long filaments when grown with ciprofloxacin, compared with wild-type strain and control groups without ciprofloxacin treatment (Fig. 5A). The length statistics of the strains in the transmission electron microscopy pictures showed that the average length of the Δ*piwi-re* strains treated with ciprofloxacin was significantly longer than that of the wild-type strains treated with ciprofloxacin (Fig. 5B). In our results, loss of function of PIWI-RE resulted in lower survival rate and long filaments after ciprofloxacin (a DNA replication inhibitor) treatment than wild-type, suggesting PIWI-RE may potentially be involved in cell division and further DNA replication. Considering ciprofloxacin target DNA gyrase and topoisomerase IV, we speculate that when the gyrase or topoisomerase IV is inhibited, *Ps*PIWI-RE can replace part of the function of gyrase or topoisomerase IV to assist in DNA replication and bacterial cell division.

Our results showed the important role of *Ps*PIWI-RE in the process of bacterial cell division, however, the molecular mechanism by which *Ps*PIWI-RE affects bacterial cell division needs more evidence. The gene coding for *Ps*PIWI-RE co-occurred with a DinG helicase and an RNase, which indicates that these proteins may function in combination with *Ps*PIWI-RE. DinG helicase is involved in DNA replication and repair (Voloshin et al. 2003; Voloshin and Camerini-Otero 2007). RNases may also contribute to the generation of guide strands. It is possible that these three proteins form a complex which functions to terminate DNA replication.

## Conclusion

For the first time, this study describes the endonuclease activity and cellular functions of the PIWI-RE family protein, *Ps*PIWI-RE. This study demonstrates that *Ps*PIWI-RE exhibits unique endonuclease activity, including RNA-guided DNA cleavage and DNA-guided RNA cleavage. Structural modelling of *Ps*PIWI-RE showed that the predicted N-terminal domain of *Ps*PIWI-RE contained a unique PAZ-like domain, in which the nucleic-acid binding fold was positioned away from the nuclease active site, a structure which is different from that of the *Pf*Ago and *Rs*Ago proteins. RNA-guided *Ps*PIWI-RE can cleave ssDNA, and the optimal temperature for this cleavage is 45 °C. A RNA guide strand of minimum length 10 nt mediates this cleavage. Asp525 and Asp610 are essential residues for this cleavage, but Arg639 and Glu718 are not. The Δ*piwi-re* strain showed high sensitivity to ciprofloxacin than wild-type strain, suggesting that *Ps*PIWI-RE can replace part of the function of gyrase to assist in DNA replication and bacterial cell division. Because the gene for *Ps*PIWI-RE is clustered with genes coding for a putative DinG helicase and a restriction enzyme, we further speculate these three enzymes work together for DNA replication or other Genomic DNA-related pathways. As an RNA-guided DNA endonuclease, *Ps*PIWI-RE is an excellent candidate for biotechnological applications, such as genome editing. It is valuable to further reveal the working mechanism of *Ps*PIWI-RE and explore its application potential in genome editing. In summary, our study sheds light on the functional role of a novel Ago subfamily in catalysis. These findings will hopefully provide a theoretical basis for the application of the PIWI-RE subfamily.

### Supplementary Information

**Additional file 1: Fig. S1**. Structure prediction of the *Ps*PIWI-RE. (A) Structure alignment of *Ps*PIWI-RE with Ago from the archaeon *Pyrococcus furiosus* (*Pf*Ago) (PDB: 1U04). (B) Structure alignment of *Ps*PIWI-RE with Ago from *Rhodobacter sphaeroides* (*Rs*Ago) (PDB: 5AWH). (C) Sequence alignment shows conserved residues in PIWI-RE proteins. (D) Magnified view of the *Ps*PIWI-RE structure with potential conserved residues highlighted. **Fig. S2**. Nucleic acid binding activity of *Ps*PIWI-RE with varying oligonucleotides determined by a fluorescence polarization assay. Titration binding curves for the *Ps*PIWI-RE protein with various 21-base nucleic acid ligands labeled with 6-FAM at the 3′ ends were obtained by increasing the concentration of *Ps*PIWI-RE: (A) 5′ T-phosphorylated-DNA; (B) 5′ U-phosphorylated-RNA; (C) 5′ T-hydroxylated DNA; (D) 5′ U-hydroxylated RNA; (E) 5′ A-hydroxylated RNA; (F) 5′ C-hydroxylated RNA; (G) 5′ G-hydroxylated RNA. The oligonucleotide sequences are shown in Supplementary Table 1. Error bars represent SD values (n = 3). **Fig. S3**. Construction of *P. stutzeri piwi-re* deletion strain (△*piwi-re*). (A) Confirmation of deletion of *piwi-re* gene in *P. stutzeri* genomic DNA. The primer pair can amplify a 2.8 kb product when using wild-type *P. stutzeri* genomic DNA (wild-type) as a template and a 500 bp product when using PIWI-RE deletion genomic DNA (△*piwi-re*). (B) Real-time PCR to confirm the knock-out of the PIWI-RE gene. cDNA from wild-type and △*piwi-re* were strains were used as templates. **Fig. S4**. Growth curve analysis of *P. stutzeri piwi-re* deletion strain (△*piwi-re*) and wild-type strain. The OD_600_ was measured every 1 hour for 16 hours. Experiments were performed in triplicate. Growth curves were plotted from triplicate data, using Graphpad GraphPad Prism 8. **Table S1**. Oligonucleotides used in this study. **Table S2**. Plasmids used in this study. **Table S3**. Proteins used in the experiments described in Figure 1A and Figure S1C. **Table S4**. DNA sequences used in this study.

## Data Availability

The authors declare that all data supporting the findings of this study are available in the paper and its supplementary information files.
