# Supplementary material for: Catalytic properties and biological function of a PIWI-RE nuclease from Pseudomonas stutzeri
Source: Bioresour Bioprocess. 2022 May 24;9(1):57. doi: 10.1186/s40643-022-00539-x (PMC10991935; doi:10.1186/s40643-022-00539-x)
Supplement: Supplementary file 1 — Additional file 1: Fig. S1. Structure prediction of the PsPIWI-RE. (A) Structure alignment of PsPIWI-RE with Ago from the archaeon Pyrococcus furiosus (PfAgo) (PDB: 1U04). (B) Structure alignment of PsPIWI-RE with Ago from Rhodobacter sphaeroides (RsAgo) (PDB: 5AWH). (C) Sequence alignment shows conserved residues in PIWI-RE proteins. (D) Magnified view of the PsPIWI-RE structure with potential conserved residues highlighted. Fig. S2. Nucleic acid binding activity of PsPIWI-RE with varying oligonucleotides determined by a fluorescence polarization assay. Titration binding curves for the PsPIWI-RE protein with various 21-base nucleic acid ligands labeled with 6-FAM at the 3′ ends were obtained by increasing the concentration of PsPIWI-RE: (A) 5′ T-phosphorylated-DNA; (B) 5′ U-phosphorylated-RNA; (C) 5′ T-hydroxylated DNA; (D) 5′ U-hydroxylated RNA; (E) 5′ A-hydroxylated RNA; (F) 5′ C-hydroxylated RNA; (G) 5′ G-hydroxylated RNA. The oligonucleotide sequences are shown in Supplementary Table 1. Error bars represent SD values (n = 3). Fig. S3. Construction of P. stutzeri piwi-re deletion strain (△piwi-re). (A) Confirmation of deletion of piwi-re gene in P. stutzeri genomic DNA. The primer pair can amplify a 2.8 kb product when using wild-type P. stutzeri genomic DNA (wild-type) as a template and a 500 bp product when using PIWI-RE deletion genomic DNA (△piwi-re). (B) Real-time PCR to confirm the knock-out of the PIWI-RE gene. cDNA from wild-type and △piwi-re were strains were used as templates. Fig. S4. Growth curve analysis of P. stutzeri piwi-re deletion strain (△piwi-re) and wild-type strain. The OD600 was measured every 1 hour for 16 hours. Experiments were performed in triplicate. Growth curves were plotted from triplicate data, using Graphpad GraphPad Prism 8. Table S1. Oligonucleotides used in this study. Table S2. Plasmids used in this study. Table S3. Proteins used in the experiments described in Figure 1A and Figure S1C. Table S4. DNA sequences used in this st [file 40643_2022_539_MOESM1_ESM.docx]

Additional file 1

**
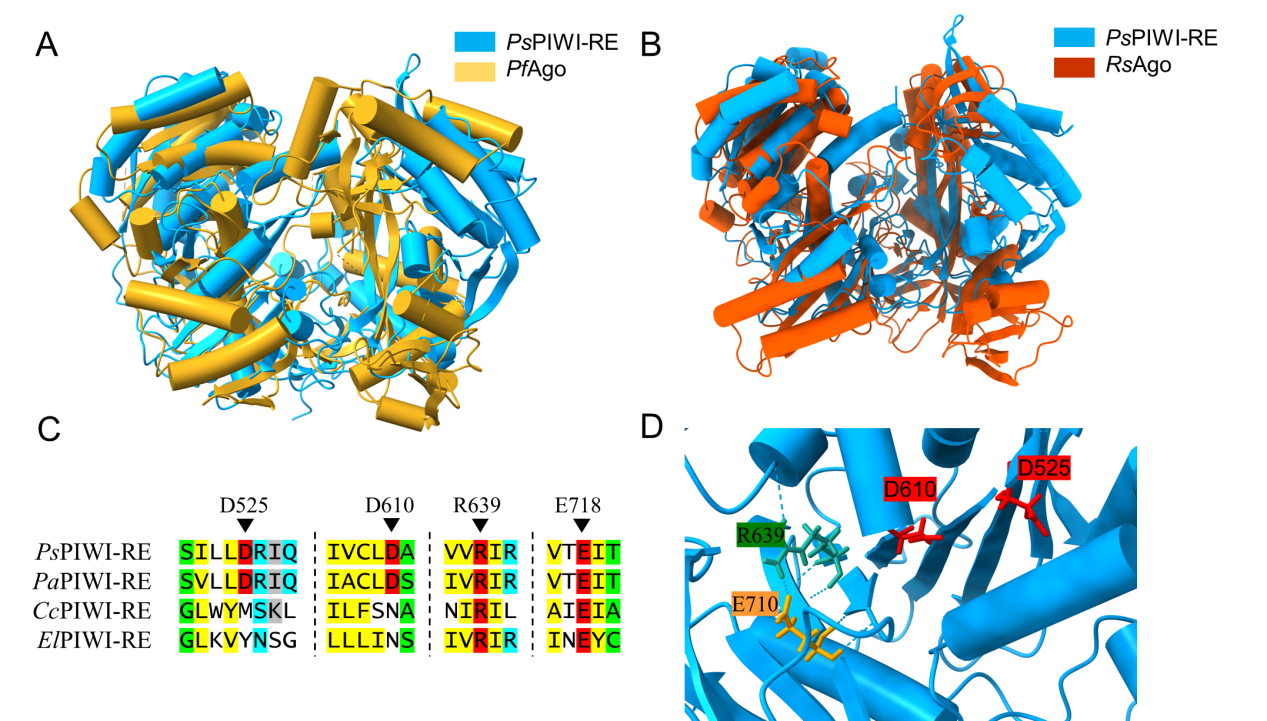
**

**Figure S1. Structure prediction of the *Ps*PIWI-RE.** (A) Structure alignment of *Ps*PIWI-RE with Ago from the archaeon *Pyrococcus furiosus* (*Pf*Ago) (PDB: 1U04). (B) Structure alignment of *Ps*PIWI-RE with Ago from *Rhodobacter sphaeroides* (*Rs*Ago) (PDB: 5AWH). (C) Sequence alignment shows conserved residues in PIWI-RE proteins. (D) Magnified view of the *Ps*PIWI-RE structure with potential conserved residues highlighted.


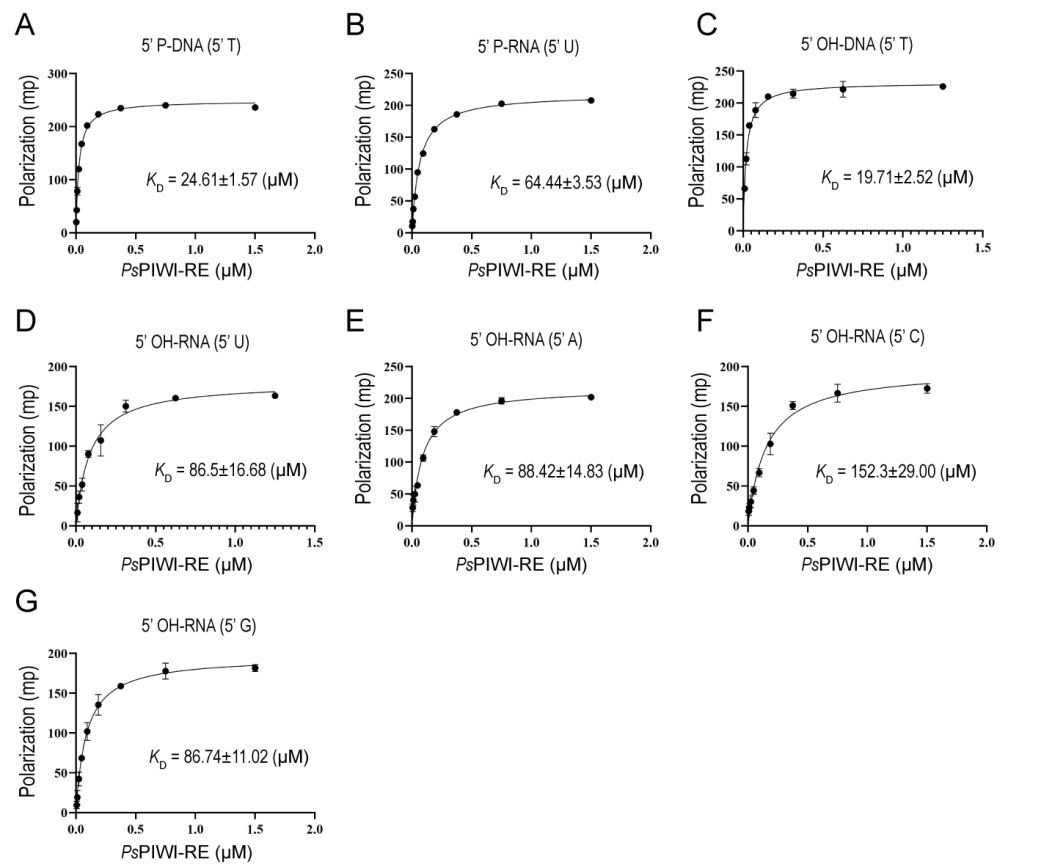


**Figure S2. Nucleic acid binding activity of *Ps*PIWI-RE with varying oligonucleotides determined by a fluorescence polarization assay.** Titration binding curves for the *Ps*PIWI-RE protein with various 21-base nucleic acid ligands labeled with 6-FAM at the 3′ ends were obtained by increasing the concentration of *Ps*PIWI-RE: (A) 5′ T-phosphorylated-DNA; (B) 5′ U-phosphorylated-RNA; (C) 5′ T-hydroxylated DNA; (D) 5′ U-hydroxylated RNA; (E) 5′ A-hydroxylated RNA; (F) 5′ C-hydroxylated RNA; (G) 5′ G-hydroxylated RNA. The oligonucleotide sequences are shown in Supplementary Table 1. Error bars represent SD values (n = 3).

**
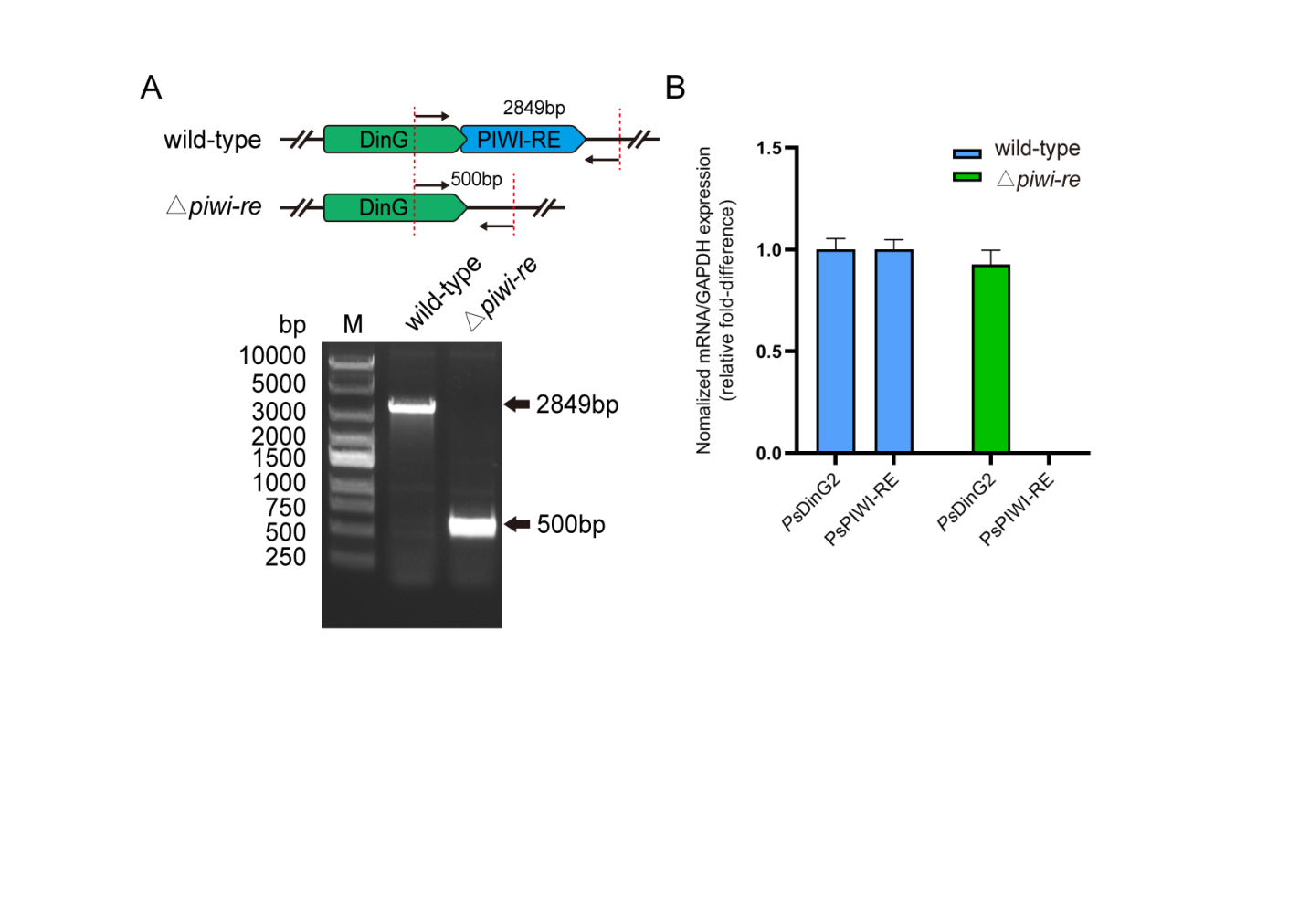
**

**Figure S3. Construction of *P. stutzeri* *piwi-re* deletion strain (△*piwi-re*).** (A) Confirmation of deletion of *piwi-re* gene in *P. stutzeri* genomic DNA. The primer pair can amplify a 2.8 kb product when using wild-type *P. stutzeri* genomic DNA (wild-type) as a template and a 500 bp product when using PIWI-RE deletion genomic DNA (△*piwi-re*). (B) Real-time PCR to confirm the knock-out of the PIWI-RE gene. cDNA from wild-type and △*piwi-re* were strains were used as templates.


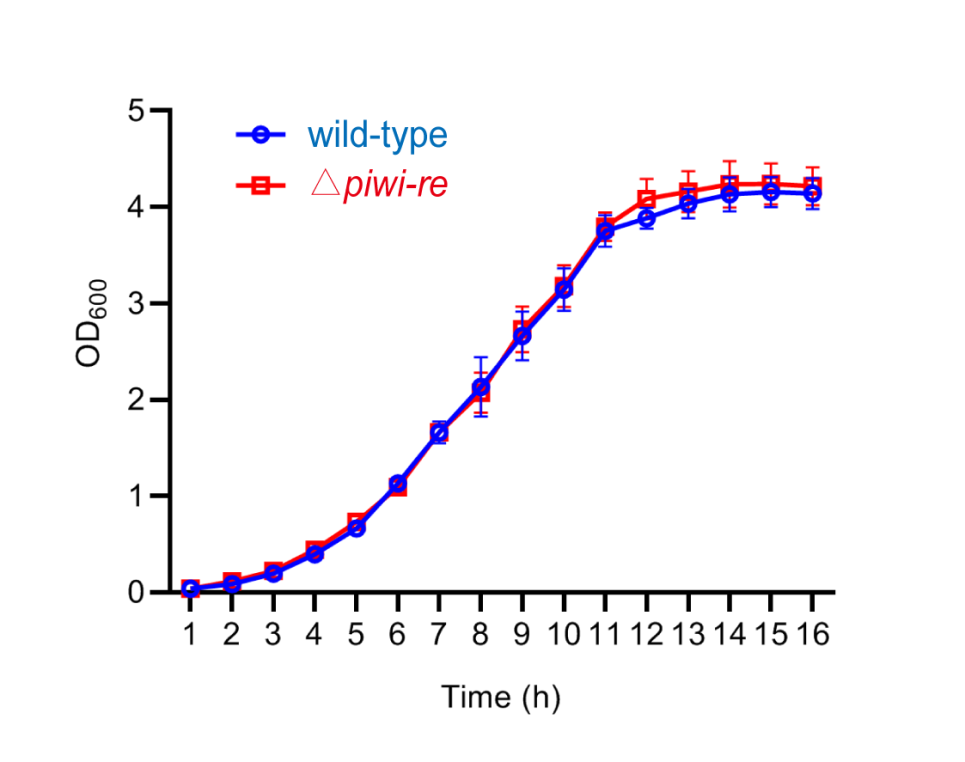


**Figure S4. Growth curve analysis of *P. stutzeri* *piwi-re* deletion strain (△*piwi-re*) and wild-type strain.** The OD_600_ was measured every 1 hour for 16 hours. Experiments were performed in triplicate. Growth curves were plotted from triplicate data, using Graphpad GraphPad Prism 8.

**Table S1.** Oligonucleotides used in this study.

| **Description** | **Sequence (5′-to-3′)** | **Experiment** |
| --- | --- | --- |
| 21-nt RNA guide with 5’P | P-UGAGGUAGUAGGUUGUAUAGU | Guide strands used in Figure 2 |
| 21-nt RNA guide with 5’OH | UGAGGUAGUAGGUUGUAUAGU |  |
| 21-nt DNA guide with 5’P | P-TGAGGTAGTAGGTTGTATAGT |  |
| 21-nt DNA guide with 5’OH | TGAGGTAGTAGGTTGTATAGT |  |
| 21-nt RNA guide with 5’P and 3’FAM | P-UGAGGUAGUAGGUUGUAUAGU-FAM | Guide strands used in Figure 1C |
| 21-nt RNA guide with 5’OH and 3’FAM | UGAGGUAGUAGGUUGUAUAGU-FAM |  |
| 21-nt DNA guide with 5’P and 3’FAM | P-TGAGGTAGTAGGTTGTATAGT-FAM |  |
| 21-nt DNA guide with 5’OH and 3’FAM | TGAGGTAGTAGGTTGTATAGT-FAM |  |
| 8-nt RNA guide with 5’OH | UGAGGUAG | Guide strands used in Figure 3A |
| 10-nt RNA guide with 5’OH | UGAGGUAGUA |  |
| 12-nt RNA guide with 5’OH | UGAGGUAGUAGG |  |
| 14-nt RNA guide with 5’OH | UGAGGUAGUAGGUU |  |
| 16-nt RNA guide with 5’OH | UGAGGUAGUAGGUUGU |  |
| 18-nt RNA guide with 5’OH | UGAGGUAGUAGGUUGUAU |  |
| 25-nt RNA guide with 5’OH | UGAGGUAGUAGGUUGUAUAGUAUAU |  |
| 31-nt RNA guide with 5’OH | UGAGGUAGUAGGUUGUAUAGUAUAUUAAAUU |  |
| 78-nt DNA target with 3’FAM | TCGTAAATAATTTAATATACTATACAACCTACTACCTCGTATAAATTTTTAAATAAATATTGCATTCAAGCTTTTAAT-FAM | Typical DNA target used in DNA cleavage assay |
| 35-nt RNA target with 3’FAM | AUAUACUAUACAACCUACUACCUCGUAUAAAUUUU | Typical RNA target used in RNA cleavage assay |
| DinG substrate F, 5’FAM | CTACTACCCCCACCCTCACAACCTTTTTTTTTTTTTT | DNA substrate used in Figure 4 |
| DinG substrate R, 3’BHQ1 | TTTTTTTTTTTTTTGGTTGTGAGGGTGGGGGTAGTAG |  |
| DinG substrate F2, 5’FAM | CUACUACCCCCACCCUCACAACCUUUUUUUUUUUUUU | RNA substrate used in Figure 4 |
| DinG substrate R2, 3’BHQ1 | UUUUUUUUUUUUUUGGUUGUGAGGGUGGGGGUAGUAG |  |
| QpsagoF | GCCTACGACAGGATTGGAGG | qPCR primers used in Figure S3 |
| QpsagoR | CCATCGACCATAGGTGGAGC |  |
| QpsdingF | TGATCCGCACAGCACTCTAC |  |
| QpsdingR | AAGGTCGTAAGCACTGGCTC |  |
| QpsnadphF | TTCGGCGTACTGCTGATCC |  |
| QpsnadphR | GTGCCGATAAAGGCGAACAC |  |
| dPSago-F1 | CGCggatccACGGTTGTTCATAGGGTTCTCTG | Primers for homology arm amplification |
| dPsAgo-R1 | GGGGTGATTCATTCCTCATCTCGCGTCCTTTCTTGATTC |  |
| dPsAgo-F2 | AAGGACGCGAGATGAGGAATGAATCACCCCGGGTTTC |  |
| dPsAgo-R2 | CCCaagcttTAGCGGATCGAGACGTACTGG |  |
| det-dPRE-F3 | TGAACATCCTGGTGAGCCTCAC | Primers used in Figure S3 |
| det-dPRE-R3 | CGCTCTTCACTGGTCTGACCG |  |

**Note:** P, monophosphate; FAM, 6-FAM; OH, hydroxyl; BHQ1, black hole quencher 1.

**Table S2.** Plasmids used in this study.

| **Plasmid** | **Description** | **Source, reference** |
| --- | --- | --- |
| pET-28a (+) | Protein expression vector | Novagen |
| pET-28a-PsPIWI-RE | Expression vector for *Ps*PIWI-RE with a N-terminal His-tag | This study |
| pK18mobSacB | Cloning vector for homologous recombination. | ATCC 87097 |
| pK18mobSacB-△*piwi-re* | Vector for *piwi-re* knock-out | This study |

**Table S3.** Proteins used in the experiments described in Figure 1A and Figure S1C.

| **Protein name** | **Strain** | **Accession number** |
| --- | --- | --- |
| *Pf*Ago | *Pyrococcus furiosus* | WP_011011654.1 |
| *Mj*Ago | *Methanocaldcoccus janaschii* | WP_010870838.1 |
| *Lr*Ago | *Limnothrix rosea* | WP_075892274.1 |
| *Cb*Ago | *Clostridium butyricum* | WP_045143632.1 |
| *Cp*Ago | *Clostridium perfringens* | EHP50500.1 |
| *Rs*Ago | *Rhodobacter sphaeroides* ATCC 17025 | ABP72561.1 |
| *Tt*Ago | *Thermus thermophilus* | WP_011174533.1 |
| *Mp*Ago | *Marinitoga piezophila* | WP_014295921.1 |
| *Dm*Ago | *Drosophila melanogaster* | NP_001261882.1 |
| *Kp*Ago | *Vanderwaltozyma polyspora* DSM 70294 | XP_001644461.1 |
| *At*Ago1 | *Arabidopsis thaliana* | NP_001185169.1 |
| hAgo1 | *Homo sapiens* | Q9UL18 |
| hAgo2 | *Homo sapiens* | Q9UKV8 |
| *Ng*Ago | *Natronobacterium gregoryi* | ANC90309.1 |
| *El*PIWI-RE | *Eggerthella lenta* DSM 2243 | YP_003181179.1 |
| *Cc*PIWI-RE | *Clostridium cellulolyticum* H10 | YP_002507007.1 |
| *Ps*PIWI-RE ★ | *Pseudomonas stutzeri* DSM 4166 | WP_014597637.1 |
| *Pa*PIWI-RE | *Pseudomonas aeruginosa* NCMG1179 | GAA16203.1 |

**Table S4.** DNA sequences used in this study.

| **Sequences** | **Description** |
| --- | --- |
| CAATGACTCGACCCTGAACGTCGTCGCCATGCGCGGTGATCCGCACAGCACTCTACTTTCACTTCCGGATGTCAGTGCTCTGGGCTATGCCGGTGTGAAACGGTTGTTCATAGGGTTCTCTGCCACTGCATATTTTCCCGGAGCCAGTGCTTACGACCTTCGAGCCAAAGACTTCATCGACGTTCCGGATGCAGCTGGCCAGATCACCTTCGAGAACGTCAACCAAACGACCGCCATCTCTGGTGCCACCTTTGCGGAGCGCAAGTTCCTGGTATCGAAATTTGCCAAAGAAATATGGCCCTGGGCGAAAGCCCGTCTCCAGAGCCTGGCAAACGACCCAAAGACCCAAGAGCGCGCTCGCCTGCTCTTGGTCACCAACAGCGATACGGATGCTGAAGTGCTGGCCATGACCCTCGCGAAAATGCAGGGCGGCCCTGGCCAATTGGTAGGCTGGGTGCGAGGACGACAGAGCGAGTACAAGCCATCCTCACTCGAAGCACAGCAAACACTTGTCTACGACGACCTGGCGGAATTCACCAGCGGCCGGCATAAGGACAAGACGTTGCTGGTCAGTGCGCTTGGCCCTATGGCGCGCGGCCATAACATCGTAAACGCCGATGGTCTTTCGGCGATTGGCGCCGTAGTGATCTGCGTCCGTCCACTGCCCTCTTCGGACAGCCCAAACAACAACCTTGCCCATATCTGCTACGAGACGGGGAAAGCGGTTGCCTTCTACAGCAGCCCTGGCTTGCTGATGATGCAGGAGCGAAAGCATTCCAATGCTCTCCTACAAAGTATTCGTACCGCACGCCCGGCGTTTAGCCAGCAACCGGATAACATCCGTCATTACACCATCATGAACATCCTGGTGAGCCTCACTCAACTCATCGGACGGGGGCGTCGAGGAGGTACTCCGGTAACTTGTTACTTCGCTGATGCCGCGTTTCTGAATGGCCTCAAGCCTTGGCACGAGATGCTCAACGAGAGCGTCCATCAACTCAAGAAGGATGGAGATTGGGATCAGTTCGAACGTCACCATGCCGGCGTCGCATCAGCACTTCTGAAATACATCAATGAATCAAGAAAGGACGCGAGATGA | The upstream homologous arms (1000 bp) used in *P. stutzeri* △*piwi-re* mutant construction. |
| AGGCCCTTGAGCTACGCACCAGCCTATTCAAATTTGATGCGACCCAATTGGGGCAAGCCTACCGTGTGGTAATCGGCCCCCAGTATCTCGACGCGTGGCAAGCGCTTCAGGGGCTGGTTAAAAAGCCACATCCGGGCCTGCCTACGACAGGATTGGAGGAGATGCTTGCCGTTCTTTCCCGGGGCCCCGTAAAGGTGGACCTATTCCCCCAAAAAAAGGGCGGTGTCTCGGCAATTCTCATGCTCTATCCGCTGTCGGTCGACACTATCAACGAGGTGCTCCACCTATGGTCGATGGACGTCCTTAGGATTTGGAACGAGCAACTGGTCGGCATTGAAGGAAAGTTGATCGTCACCGACGTGGTGCCGCTGGATACAAGTCGTCTAGTCACGCCTGGAGACATTTCATCGCTCGCGTATACGGTCATTCCTTGGCTGGTGGGACAAGCCCTCATACAAACGCCCATGCAGGCAGCGAGGCCCATCAAGCTGTACCAGGCAGCTGATTCCAGCTTGCTTGCATGGGATGACCCTATCGTTTCCGAAAATGATGTCCGATATGCCAGTGCACTGCATGCTATCGAACCGACTCTTGTCTTGCTGCACGGTCGGCCGCAGCCCTACATCCAGCTACGTGTGAAACTGACCCAGGTGATGCCCAACCTTGTAGGCAAGAAAAAACATGCCTGGGTCAAAACCGGCGACCTGATCGTCAAAGCGAAGCTCAAAACCAAGAAGACGGACGAAGGCTGGGAAACTACGTACGAGCACCCTGTCGAGAAGCTGCTGACCTTCATGGGCGTACAGTCGTTTCCTCCAATGGTCGACGGCGACATCCCCGTCGACAGCGACGTGAGACCCATCTACGCCATCCCACCGTCAAATCCAATGATTGCGTCAGGCCCTGGCCCGCTGTTCCTCGACCAAGCCGGCTTTCACCTCCTTGCAAGTCTGCCTGGAACGGCTCCGCTTCTGGTCAAGAAGGCGGTGGCTAGCTTACGAGAGGAGAAGGTTGTCAACACGGGAGAGGCCGCCAATCTGAACGCGATGGTACTCGCAGCACACGCTGACGTGATGCTGCGGCTACATGCAGCCAGTACCACCCTGGCCCAGGACAGCAAGTTCTTCGATAAGGTGATGCCTCCTCTCGTGGCACTGACACGCCTGGATGTACCGGACGCGCAGCGAATGCTTGAAGGAAAACATGACAGCAACAGCCTAAACGACTGGCTAATGAACCATGTGGTGCCCGCTAGCAAGCAGGCTTCTGAAAATGGCGCAAAGGTAATGATCGTTGAGACCAGTACGTCCGCTGCCTCTCAGGAAGCAGGGCTAGACCCCAAGCACGTCATTCGAAGGGTACTGGCAAAGCACGGCATCGCGACCCAGTTCATCATGCATATCGACCCTGATGCACAAGCTAAGAGGCGGAAGACCAAAGCGGATGATCGTGATTTCAAAGCTACCAACTCGATCATCGAAGCGATTCGACTAAGCGGGCACCTCCCCGTTCCGACGCCCAAAGTAAAATCGATGCCGGCGAGCACAACGGTGCTGTCGATTTTGCTGGATAGAATTCAGGATAAAGGCCCGGCCATCTATCTGCCGGTTATCACCCGGACGGTGTTGGGCGGGAATAAACCAGAGGTTTTCTGGTTCGAATCTTGCTTGGACTCCAATGGCAAATGGTTCAGCTACGGCGAGGGCTTGGCCGCCATCCACGGGACGGACACTCTGCTCAAGCCTGACCAATTGAAGACATTGGTCACCCAATCCTTGCTGGACTGCAAGATCAATTCGAACGACTCGTTGATCGTCTGCCTCGATGCCAATCTAAGAACCTTCTATGGAGCATTGAAAGATGGCCCCGGGGAGGGTCTTCCTCCCGTCCCATCAGATGCCGCTGTCGTCCGCATTCGAGCCGATCACCAGGTAGCACAGATCAGCGGCAACCACACCTTGTCTCCCAACTCGGCTCACTACATCGGGACGAAGGTGGGAGCTTTTCAATCTTGCGAGAGCGCCTCGGTATTTTATTTCGTGTCACCGTCTAAGCAGTACGGCAGCGTTCGCTCACAGCGCGAGAACACAAGATACGACGTATCAGAACGAGACCTGCGAGATCCATGGCAGCAGTTGGGCGTAACAGAAATCACGATCATAACGCCCGGGGCATTTAGCACTGCGACAGTGATCGCTGAACAGGTCGCCTTGCTGTGCAGGAACCCTTCACTGTGGGACGGCTACCTGCGTCTGCCTGGGCCCATGCACTTGGGCAAACAAGTAGCGGCAGACCATCCAATTTTGGAAATGCGACGCAAGTCTGAAGCGAACCGGTATGGAAATTAG | The deletion region, inclouding the entire coding region (except nucleotides 1-4) of *piwi-re* gene. |
| GGAATGAATCACCCCGGGTTTCGTGGAGGCCTCAACTCTTGAGAAGATGAGGCCATGAGAAAGACTACTACCTACTCCCCTGAAGTCCGTGAGCGTGCTGTGCGCATGGTTCTGGAACACCTGAACGACTATCCGTCCGAGTGGGCAGCCATTGAGGCCATCGCTCCGAAGATTGGCTGTGCCGCGCAAACCCTGCATGGCTGGATTCGTCGCCAGCAGACCGATGCGGGGCAGCGCCCCGGTCAGACCAGTGAAGAGCGCGAGCGCATCAGAGCCCTAGAGCGCGAAAACCGCGAACTGCGTAAGGCAAACGAGATATTGCGCCTGGCCAGTGCGTATTTTGCCCAGGCGGAGCTCGACCGCCGCACCAAGTCCTGAGGGCGTTTGTCGATCAGCATCGTGACCGTCTCGGGGTCGAGTCGATCTGCCGCGTGTTGCAGATCGCCCCGTCCGGTTACCGCAGGCACGTGGCTCAACAGCGCAACCCGGCACTGCGCTGTTGTCGTGCTCAGCGCGATGACGCATTGACCCTGGAAATCCAGCGAGTGTGGGATGCCAATATGCAGTGCTATGGCGCGGTGAAGGTCTGGAAGCAGCTGCGGCGAGAAGGCATCGAGGTCGCCAGATGCACGGTGGAGCGGTTAATGCGTCGGGCCGGATTGCAGGGCATTAGACGTGGCCAGATCGTGCAGACAACGGTGGCCGGCGACAAGGCCCTTTGCCCGCTGGATCGTGTCCAACGCCAGTTCCATGCCGACCGCCCGAACCAGTTGTGGGTGTCGGACTTCACCTATGTATCGACCTGGCAGGGCTGGCTGTACGTGGCGTTCGTGATCGACGTCTTTGCACGGCGGATCGTCGGCTGGCGAGTCAGTACCAGCATGAAGACAGACTTCGTACTGGATGCCCTGGAGCAGGCCCTGTACGCCCGCCAGCCACACCGTACCGGTGGTCTGATCCATCACAGCGACCGTGGAAGCCAGTACGTCTCGATCCGCTATACCGAACGGCTGGCAGAGGCCGGCATTGAGCC | The downstream homologous arms (1000 bp) used in *P. stutzeri* △*piwi-re* mutant construction. |
| >NC_017532.1:3802322-3804673 Pseudomonas stutzeri DSM 4166  ATGAAGGCCCTTGAGCTACGCACCAGCCTATTCAAATTTGATGCGACCCAATTGGGGCAAGCCTACCGTG  TGGTAATCGGCCCCCAGTATCTCGACGCGTGGCAAGCGCTTCAGGGGCTGGTTAAAAAGCCACATCCGGG  CCTGCCTACGACAGGATTGGAGGAGATGCTTGCCGTTCTTTCCCGGGGCCCCGTAAAGGTGGACCTATTC  CCCCAAAAAAAGGGCGGTGTCTCGGCAATTCTCATGCTCTATCCGCTGTCGGTCGACACTATCAACGAGG  TGCTCCACCTATGGTCGATGGACGTCCTTAGGATTTGGAACGAGCAACTGGTCGGCATTGAAGGAAAGTT  GATCGTCACCGACGTGGTGCCGCTGGATACAAGTCGTCTAGTCACGCCTGGAGACATTTCATCGCTCGCG  TATACGGTCATTCCTTGGCTGGTGGGACAAGCCCTCATACAAACGCCCATGCAGGCAGCGAGGCCCATCA  AGCTGTACCAGGCAGCTGATTCCAGCTTGCTTGCATGGGATGACCCTATCGTTTCCGAAAATGATGTCCG  ATATGCCAGTGCACTGCATGCTATCGAACCGACTCTTGTCTTGCTGCACGGTCGGCCGCAGCCCTACATC  CAGCTACGTGTGAAACTGACCCAGGTGATGCCCAACCTTGTAGGCAAGAAAAAACATGCCTGGGTCAAAA  CCGGCGACCTGATCGTCAAAGCGAAGCTCAAAACCAAGAAGACGGACGAAGGCTGGGAAACTACGTACGA  GCACCCTGTCGAGAAGCTGCTGACCTTCATGGGCGTACAGTCGTTTCCTCCAATGGTCGACGGCGACATC  CCCGTCGACAGCGACGTGAGACCCATCTACGCCATCCCACCGTCAAATCCAATGATTGCGTCAGGCCCTG  GCCCGCTGTTCCTCGACCAAGCCGGCTTTCACCTCCTTGCAAGTCTGCCTGGAACGGCTCCGCTTCTGGT  CAAGAAGGCGGTGGCTAGCTTACGAGAGGAGAAGGTTGTCAACACGGGAGAGGCCGCCAATCTGAACGCG  ATGGTACTCGCAGCACACGCTGACGTGATGCTGCGGCTACATGCAGCCAGTACCACCCTGGCCCAGGACA  GCAAGTTCTTCGATAAGGTGATGCCTCCTCTCGTGGCACTGACACGCCTGGATGTACCGGACGCGCAGCG  AATGCTTGAAGGAAAACATGACAGCAACAGCCTAAACGACTGGCTAATGAACCATGTGGTGCCCGCTAGC  AAGCAGGCTTCTGAAAATGGCGCAAAGGTAATGATCGTTGAGACCAGTACGTCCGCTGCCTCTCAGGAAG  CAGGGCTAGACCCCAAGCACGTCATTCGAAGGGTACTGGCAAAGCACGGCATCGCGACCCAGTTCATCAT  GCATATCGACCCTGATGCACAAGCTAAGAGGCGGAAGACCAAAGCGGATGATCGTGATTTCAAAGCTACC  AACTCGATCATCGAAGCGATTCGACTAAGCGGGCACCTCCCCGTTCCGACGCCCAAAGTAAAATCGATGC  CGGCGAGCACAACGGTGCTGTCGATTTTGCTGGATAGAATTCAGGATAAAGGCCCGGCCATCTATCTGCC  GGTTATCACCCGGACGGTGTTGGGCGGGAATAAACCAGAGGTTTTCTGGTTCGAATCTTGCTTGGACTCC  AATGGCAAATGGTTCAGCTACGGCGAGGGCTTGGCCGCCATCCACGGGACGGACACTCTGCTCAAGCCTG  ACCAATTGAAGACATTGGTCACCCAATCCTTGCTGGACTGCAAGATCAATTCGAACGACTCGTTGATCGT  CTGCCTCGATGCCAATCTAAGAACCTTCTATGGAGCATTGAAAGATGGCCCCGGGGAGGGTCTTCCTCCC  GTCCCATCAGATGCCGCTGTCGTCCGCATTCGAGCCGATCACCAGGTAGCACAGATCAGCGGCAACCACA  CCTTGTCTCCCAACTCGGCTCACTACATCGGGACGAAGGTGGGAGCTTTTCAATCTTGCGAGAGCGCCTC  GGTATTTTATTTCGTGTCACCGTCTAAGCAGTACGGCAGCGTTCGCTCACAGCGCGAGAACACAAGATAC  GACGTATCAGAACGAGACCTGCGAGATCCATGGCAGCAGTTGGGCGTAACAGAAATCACGATCATAACGC  CCGGGGCATTTAGCACTGCGACAGTGATCGCTGAACAGGTCGCCTTGCTGTGCAGGAACCCTTCACTGTG  GGACGGCTACCTGCGTCTGCCTGGGCCCATGCACTTGGGCAAACAAGTAGCGGCAGACCATCCAATTTTG  GAAATGCGACGCAAGTCTGAAGCGAACCGGTATGGAAATTAG | DNA sequence of *Ps*PIWI-RE |
| ATGAAGGCGCTGGAACTGCGTACCAGCCTGTTCAAATTTGATGCGACCCAGCTGGGCCAAGCGTACCGTGTGGTTATTGGTCCGCAGTATCTGGATGCGTGGCAGGCGCTGCAAGGTCTGGTTAAGAAACCGCATCCGGGTCTGCCGACCACCGGTCTGGAGGAAATGCTGGCGGTGCTGAGCCGTGGTCCGGTGAAGGTTGACCTGTTCCCGCAAAAGAAAGGTGGCGTTAGCGCGATCCTGATGCTGTACCCGCTGAGCGTTGATACCATTAACGAGGTGCTGCACCTGTGGAGCATGGACGTGCTGCGTATCTGGAACGAGCAGCTGGTTGGCATCGAAGGCAAGCTGATTGTGACCGACGTGGTTCCGCTGGATACCAGCCGTCTGGTGACCCCGGGCGACATCAGCAGCCTGGCGTACACCGTGATTCCGTGGCTGGTTGGTCAGGCGCTGATCCAAACCCCGATGCAGGCGGCGCGTCCGATTAAACTGTATCAAGCGGCGGATAGCAGCCTGCTGGCGTGGGACGATCCGATCGTGAGCGAAAACGACGTTCGTTATGCGAGCGCGCTGCATGCGATTGAACCGACCCTGGTGCTGCTGCATGGTCGTCCGCAACCGTATATCCAGCTGCGTGTGAAGCTGACCCAGGTTATGCCGAACCTGGTGGGCAAGAAAAAGCACGCGTGGGTTAAAACCGGTGATCTGATCGTGAAAGCGAAGCTGAAAACCAAAAAGACCGACGAGGGTTGGGAAACCACCTACGAGCACCCGGTTGAAAAACTGCTGACCTTCATGGGCGTTCAAAGCTTTCCGCCGATGGTGGACGGTGATATTCCGGTGGACAGCGATGTTCGTCCGATCTATGCGATTCCGCCGAGCAACCCGATGATTGCGAGCGGTCCGGGTCCGCTGTTCCTGGATCAAGCGGGTTTTCACCTGCTGGCGAGCCTGCCGGGTACCGCGCCGCTGCTGGTGAAAAAGGCGGTTGCGAGCCTGCGTGAGGAAAAGGTGGTTAACACCGGTGAAGCGGCGAACCTGAACGCGATGGTGCTGGCGGCGCATGCGGATGTGATGCTGCGTCTGCATGCGGCGAGCACCACCCTGGCGCAAGACAGCAAGTTCTTTGATAAAGTTATGCCGCCGCTGGTTGCGCTGACCCGTCTGGATGTGCCGGATGCGCAGCGTATGCTGGAGGGCAAACACGACAGCAACAGCCTGAACGATTGGCTGATGAACCACGTGGTTCCGGCGAGCAAGCAAGCGAGCGAGAACGGTGCGAAAGTGATGATTGTTGAAACCAGCACCAGCGCGGCGAGCCAGGAAGCGGGTCTGGACCCGAAGCACGTGATCCGTCGTGTTCTGGCGAAACACGGTATTGCGACCCAATTCATCATGCACATTGACCCGGATGCGCAGGCGAAGCGTCGTAAGACCAAAGCGGACGATCGTGACTTTAAAGCGACCAACAGCATCATTGAAGCGATCCGTCTGAGCGGTCACCTGCCGGTTCCGACCCCGAAGGTGAAAAGCATGCCGGCGAGCACCACCGTGCTGAGCATCCTGCTGGACCGTATTCAGGATAAGGGTCCGGCGATCTACCTGCCGGTTATTACCCGTACCGTGCTGGGTGGCAACAAGCCGGAAGTGTTCTGGTTTGAAAGCTGCCTGGATAGCAACGGCAAATGGTTCAGCTATGGTGAAGGCCTGGCGGCGATTCACGGTACCGACACCCTGCTGAAGCCGGATCAACTGAAAACCCTGGTTACCCAGAGCCTGCTGGACTGCAAGATCAACAGCAACGATAGCCTGATTGTGTGCCTGGACGCGAACCTGCGTACCTTTTATGGTGCGCTGAAAGATGGTCCGGGTGAAGGTCTGCCGCCGGTTCCGAGCGATGCGGCGGTGGTTCGTATCCGTGCGGACCACCAGGTGGCGCAAATTAGCGGTAACCACACCCTGAGCCCGAACAGCGCGCACTACATCGGCACCAAGGTTGGTGCGTTCCAGAGCTGCGAGAGCGCGAGCGTGTTCTACTTTGTTAGCCCGAGCAAACAATATGGTAGCGTTCGTAGCCAGCGTGAGAACACCCGTTATGACGTGAGCGAACGTGACCTGCGTGATCCGTGGCAGCAACTGGGCGTGACCGAAATCACCATCATTACCCCGGGTGCGTTTAGCACCGCGACCGTTATTGCGGAGCAAGTGGCGCTGCTGTGCCGTAACCCGAGCCTGTGGGATGGTTACCTGCGTCTGCCGGGTCCGATGCACCTGGGCAAGCAGGTTGCGGCGGACCACCCGATCCTGGAGATGCGTCGTAAAAGCGAAGCGAACCGTTATGGTAACTAA | Optimized DNA sequence of *Ps*PIWI-RE |
